# Exploring Oxidoreductases from Extremophiles for Biosynthesis in a Non-Aqueous System

**DOI:** 10.3390/ijms24076396

**Published:** 2023-03-29

**Authors:** Shizhen Wang, Hangbin Lei, Zhehui Ji

**Affiliations:** 1 Department of Chemical and Biochemical Engineering, College of Chemistry and Chemical Engineering, Xiamen University, Xiamen 361005, China; 2 Xiamen Key Laboratory of Synthetic Biotechnology, Xiamen University, Xiamen 361005, China

**Keywords:** extreme oxidoreductases, non-aqueous system, conservation, co-evolution analysis, Interaction network

## Abstract

Organic solvent tolerant oxidoreductases are significant for both scientific research and biomanufacturing. However, it is really challenging to obtain oxidoreductases due to the shortages of natural resources and the difficulty to obtained it via protein modification. This review summarizes the recent advances in gene mining and structure-functional study of oxidoreductases from extremophiles for non-aqueous reaction systems. First, new strategies combining genome mining with bioinformatics provide new insights to the discovery and identification of novel extreme oxidoreductases. Second, analysis from the perspectives of amino acid interaction networks explain the organic solvent tolerant mechanism, which regulate the discrete structure-functional properties of extreme oxidoreductases. Third, further study by conservation and co-evolution analysis of extreme oxidoreductases provides new perspectives and strategies for designing robust enzymes for an organic media reaction system. Furthermore, the challenges and opportunities in designing biocatalysis non-aqueous systems are highlighted.

## 1. Introduction

What makes oxidoreductases particularly interesting from a standpoint of industrial application is their functional complementarity for the biosynthesis of chiral fine chemicals and application in biosensors [1]. Non-aqueous biocatalysis can potentially improve bioprocess efficiency and economics. Great achievements of non-aqueous biocatalysis have been obtained with hydrolases, namely, lipases, esterases, cellulases, etc. However, compared with the extensive study of hydrolases in non-aqueous systems, very few oxidoreductases have been reported. The lack of a broad range of robust oxidoreductases for non-aqueous biocatalysis is hindering the full potential of biotransformation. Developing enzymes in a non-aqueous system for industrial application is a strong drive to bridge the gap between lab and industrial scale [2].

Extremophiles are found in the most severe environments, such as hydrothermal vents, hypersaline lakes, alkaline soda pools, and volcanic areas [3]. To survive in such extreme conditions, extremophiles employ a variety of adaptive strategies, such as the production of extremozymes, which exhibit outstanding thermal or cold adaptability, salt tolerance, or pressure tolerance. Novel extreme oxidoreductases with industrial potential are sustainable alternatives for the chemical industry. 

Several in-depth reviews have been published recently about extremozymes [4]. Wang et al. [5] summarized microbial halophilic lipases, which were collected over the last decades on halophilic lipase sources and advances. Ando et al. [6] published a review, in particular studying the molecular basis for extreme enzymes. Arshi et al. [7] summarized the unique characteristics of halophilic proteins with highly acidic and hydrophilic residues, which make the halophilic proteins soluble and fold reversibly.

**Figure 1:**
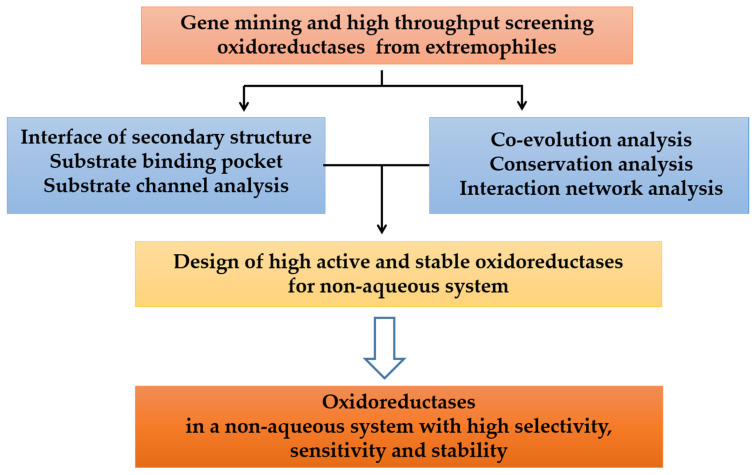
Exploring and designing oxidoreductases from extremophiles for biocatalysis in a non-aqueous system.

Biocatalysis in high-concentration organic solvents offers many advantages, which include effective solvation of reactants, minimization of substrate or product inhibition, and easy separation. However, realizing this process remains a huge challenge. The development of biocatalysts for non-aqueous systems involves the adaption of the entire protein structures, which may decrease the catalytic efficiencies. In this paper, we focus on reviewing organic solvent tolerant oxidoreductases from the viewpoints of gene mining, structure-function characterization, and then the design principle of enzymes for non-aqueous system (Figure 1). First, bioinformatic tools for functional clustering and prioritization of promising sequences from enormous genome/protein databases were introduced. Second, recently reported reaction catalyzed by oxidoreductases in organic media was summarized. Third, the structure-function analysis of extreme oxidoreductases is addressed from the perspectives of amino acid residues interaction networks and molecular dynamic simulation (MDS). Conserved and co-evolving amino acids unveil structural domains and dynamical interactions. Forth, the design of novel oxidoreductases based on the conservation and co-evolution analysis was proposed. Finally, the challenges of developing novel industrial biosynthesis routes by extremozymes in non-aqueous system are addressed. 

## 2. Mining Oxidoreductases from Extremophiles

The mining of organic solvent tolerant oxidoreductases is a challenge due to the limited natural habitat of organic solvents. Environments with low water activity, such as in a brine and marine, host thriving populations of halophilic enzymes, which provide organic solvent tolerant enzymes [8]. The diversity of extremophilic microorganisms and their enzymes represents a database that can be exploited for biotechnological and industrial applications requiring enzymes with specialized properties. The benefits from a high throughput platform for cloning genes, protein expression, and characterization of selected candidates have been explored. Jennifer et al. [9] reported halophilic alcohol dehydrogenase from the *Haloquadratum walsbyi* (HwADH), which was active in the presence of 10% (*v*/*v*) organic solvents (acetone, ACN, MeOH and iPrOH). The enzyme displayed NAD^+^/NADP^+^ dual cofactor specificity and a broad substrate scope, including benzyl alcohol and 2-phenyl-1-propanol, acetophenone, and phenylacetone. Alsafadi et al. [10] reported halophilic alcohol dehydrogenase (ADH2) from *Haloferax volcanii,* which was remarkably stable in aqueous-organic medium containing dimethyl sulfoxide (DMSO) and methanol (MeOH). Ding et al. [11] reported amino acid dehydrogenase (LsGDH) from *Lysinibacillus sphaericus* G10, which incubated at room temperature (25 °C) for 1 h and retained 101.0% activity in DMSO (60%), 73.7% activity in *n*-Hexane (60%), and 90.7% activity in *n*-Pentanol (60%). Xi et al. [12] reported a novel thermostable alcohol dehydrogenase (ADH) identified from the hyperthermophilic archaeon. The enzyme showed high resistance to organic solvents and was particularly highly active in the presence of 20% 2-propanol and 50% n-hexane or n-octane.

The rapid development of technologies of gene sequencing and bioinformatics has accumulated huge databases for genomics. This treasure trove of data provides us a wealth of research materials. Therefore, an effective tool for biological data processing is required [13]. Using the metagenomics approaches to characterize an extreme environment is transforming our view of microbial communities and their biodiversity [14,15]. Our group applied bioinformatic identification combined with structure classification, which led to 17 candidate genes for amino acid dehydrogenase. Further evaluation of thermodynamic parameters narrowed candidates down to a thermophilic and halophilic phenylalanine dehydrogenase from *Natranaerobius thermophilus.* This enzyme activity for the synthesis of L-homophenylalanine by reductive amination was enhanced by 1.2-fold with 30% DMSO [16]. Luca et al. [17] extended the library of NADH dependent light-dependent protochlorophyllide oxidoreductases (LPORs). The selected LPORs originating from *Thermosynechococcus elongatus* BP-1 contained a conserved NADPH-binding motif and three to four conserved cysteine residues that are most likely involved in substrate binding and catalysis mechanism. LPORs had 20% activity activation in 30 % DMSO, which indicated good organic solvent tolerance.

A high throughput screening strategy of organic solvent-tolerant oxidoreductases from extremophiles was developed for exploring the organic solvent-resistant amino acid dehydrogenases and amine dehydrogenases from marine bacteria [18]. Furthermore, an co-operated work integrated microbial resource for the curation and comparative analysis of genomic and metabolic data was developed for amine dehydrogenases exploring [19].

Extreme environments allow for the investigation of life’s capacity and limitations to cope with far-from-average environmental conditions in the industry. Tracing the evolution of enzyme activity and stability from extreme organisms illustrates the active pressure versus passive drift in evolution on a molecular level, refuting the activity/stability trade-off. The catalytic speed of halophilic and thermophilic oxidoreductases is an evolutionary driver for organismal fitness. Natural selection drives adaptive evolution and induces speciation. The integration of genome annotation and analysis of genomic and structural features are also to the benefit of understanding their mechanism of resistance to organic solvents [20]. From gene mining, structure characterization and function recognition to design and innovation provide a new strategy for deep mining of oxidoreductases from extremophiles and lay the foundation for designing and assembling robust oxidoreductases. Combined with multiple mining methods, the advances of enhancing the accuracy and scope of modelling and simulation in a short time frame are promoted. In addition, this strategy will yield new insights into how the adaptation to environmental stress leads to hybrids between tolerant and susceptible populations, and how they differ from their parents. 

## 3. Reaction of Oxidoreductases in Organic Media

Biocatalysis in organic solvents are important for the synthesis of chiral intermediates for pharmaceuticals, flavors, and fragrances. For oxidoreductases, the asymmetric reduction of hydrophobic and bulky substrates still remain a challenge due to the low solubility of substrate and steric hindrance effect. Despite these advantages, native oxidoreductases almost universally exhibit low activities and stabilities in a non-aqueous system. This is because oxidoreductases have naturally evolved to catalyze in an aqueous system [21].

Enzymes can catalyze valuable biochemical reactions. Their proper functioning is highly dependent on their 3D structure and stability, which is affected by their local environment. Despite their critical importance to biotechnology, there is limited knowledge of how the stability of enzymes is affected by the non-aqueous system. Recently reported oxidation-reduction reactions that are catalyzed by oxidoreductases in organic media are summarized and listed in Table 1.

The key role of organic solvents is to regulate enzymes activity by balancing the surface charge, and also accelerate hydrophobic substrates diffusion. Saadet et al. [24] studied the formate dehydrogenase (BdFDH), which displays dual coenzyme specificity and a resistance to DMSO. Martina et al. [22] reported a new alcohol dehydrogenase (HeADH-II) which was stable in polar organic solvents. It was mined from the genome of the halo bacteria. Shikha et al. [25] reported that glucose-1-dehydrogenase (GDH) from *Paenibacillus pini* was tolerant to organic solvents, as it retained 60% activity in the presence of 50% (*v*/*v*) *n*-butanol. The mechanistic understanding of the entire organic solvents-reaction system can provide valuable guidance and a general methodology for enzyme design.

## 4. Mechanism Study of Extreme Oxidoreductases in Organic Solvents

### 4.1. Structural and Dynamic Analysis 

Proteins are generally packed with a hydrophobic core and potential charged stabilizing motifs. Most of the denaturation of enzymes is initiated by the diffusion of organic solvents into a hydrophobic core. The denaturation is induced by a collapse of the hydrophobic core with organic solvents [28]. Weak interactions, such as hydrogen bonds, hydrophobic interactions, and salt bridges, stabilize the tertiary structure and play key roles in enzyme tolerance against both polar and non-polar organic solvents. Research demonstrated that the enzyme hydration shell is the deciding and independent factor determining the enzyme’s organic solvents resistance. Although it was found that halophilic enzymes can be tolerant against organic solvents, the structural basis is relatively unrevealed [28]. Current structural studies of salt-tolerant enzymes are normally based on the analysis of statistical characteristics such as amino acid composition, charged residues content, average hydrophobic fraction and B-factor. Nevena et al. [29] studied the activity, stability, and enantioselectivity of Halohydrin dehalogenases (HHDH) from *Agrobacterium radiobacter* AD1 (HheC), which was evaluated in various aqueous-organic media. A correlation was discovered between the enzyme activity in the ring-closure reaction and LogP of the solvent. Hydrophilic solvents have a considerably negative effect on enzyme activity compared to hydrophobic ones. HheC performs better in the presence of hydrophobic solvents rather than hydrophilic solvents. The coupling of dynamics to enzyme function such as tunneling on the enzyme rate is important. How mutations alter the molecular architecture of enzymes to enhance their organic solvents tolerance is the key question. Researchers have attempted to find the real general mechanisms that are common to most proteins. The organic solvents bind to the enzyme surface and can be considered as interfacial toxicity related to the functional groups of the solvent [30]. Furthermore, some organic solvents tend to access the active sites of the enzyme and affect the enzymatic performance. Halophilic proteins improve stability with an abundance of acidic residues. Samuel et al. [31] reported the structural evidence for solvent-stabilization by aspartic acid as a mechanism for the stability of halophilic enzyme in high salt concentrations. Li et al. [32] summarized the structures of alcohol dehydrogenases and reductases that crystallized in organic-aqueous systems using a structure from the Protein Data Bank. The structure-based mechanisms that are tolerant towards organic solvents were studied by comparing the structural assignments of alcohol dehydrogenases. Vygailė et al. [33] studied the effect of organic solvents on the activity and structure of glucose oxidase from *Aspergillus niger* by using chiroptical spectroscopy and molecular dynamics simulations. They showed that apolar solvents reduce the enzyme activity as they facilitate its aggregation through inter-enzymatic salt bridges.

Internal motions and conformational fluctuations can explain the dynamic aspects of the enzyme for organic solvent tolerance. Multi-level structural blocks of the enzymes (from individual residues to motifs and full domains) undergo concerted motions on a wide range of time-scales. The stability of the organic solvent-tolerant enzyme is more related to the dynamic conformation of the three-dimensional rearrangement of the amino acid residues [34]. By molecular dynamics simulations (MDS), the dynamic structural behavior of enzymes can be investigated. MDS analysis reveals protein-wide as well as local adaptation in non-aqueous media. A detailed understanding of the molecular determinants of enzyme behavior corresponding to non-aqueous media from an energy perspective is required, as each amino acid residue is essential to illuminate the energy change induced by media change. The results are interpreted with MDS by assessing the organic solvent distribution, and the enzyme dynamic conformation change [35]. Zhang et al. [30] explored the effects of organic solvents on horse liver alcohol dehydrogenase (HLADH) by combining experimental and molecular dynamics simulations. The catalytic performance of HLADH concerning its activity, stability, and selectivity is experimentally evaluated. The results are interpreted with molecular dynamics simulations by assessing the protein location in biphasic media, organic solvent distribution, and enzyme conformation. The data showed that methyl tert-butyl ether was an optimal option for the enzyme.

The benefits of considering dynamics as an integral part of the enzyme function can also enable engineering enzymes for industrial and medicinal applications [36]. It is also critical to understanding how the organic solvent properties, such as polarity and hydrophobicity, influence the enzyme dynamic conformation. New enzyme functions often evolve through the recruitment and optimization of enzyme activities.

### 4.2. Amino Acid Residues Interaction Network

The amino acid interaction network explains the highly connected structural features of the enzyme [37]. The analysis of the amino acid networks via network centrality and cluster coefficients led to identifying hot amino acid residues that play key functional roles in the conformational response to organic solvents. The network properties of extreme enzymes that are sensitive to conformational changes in non-aqueous system remain to be addressed. Further understanding of the extreme enzyme structure from the perspective of residue interaction networks (RIN) indicates that there are more salt bridges than the ordinary enzyme. Jiang et al. [38] reported a solvent-tolerant isopropanol dehydrogenase (IDH) from *Aspergillus fumigatus* Af293. The enzyme exhibits high catalysis ability of methanol, ethanol, ethylene glycol, glycerol, isopropanol, *n*-butanol, isobutanol and acetone. It also obtains high activity in organic solvents, such as isopropanol, acetonitrile, acetone, and acetophenone.

**Figure 2:**
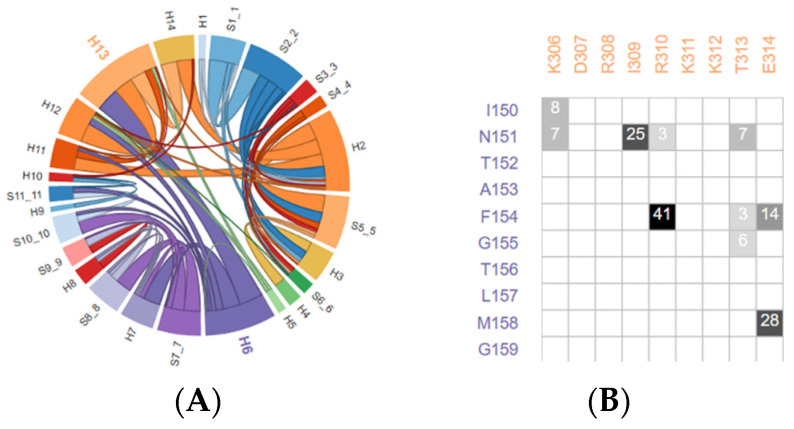
(**A**) Interfacial interactions of the secondary structure of phenylalanine dehydrogenase from *Natranaerobius thermophilus* by Protein Contacts Atlas; (**B**) Interactions heatmap of HELIX6-HELIX13 interactions.

These networks are hypothesized to serve as pathways of the energy transfer that enables thermodynamical coupling of the organic solvents with enzyme catalysis and play a key role to promote the function of the enzyme. The interfacial interaction network of the secondary structure of extreme oxidoreductases can be studied on the webserver of the Protein Contacts Atlas [39]. The interfacial interaction network of A and B subunits of phenylalanine dehydrogenase from *Natranaerobius thermophilus* was investigated (Figure 2A). The interactions of each interfacial residue can also be calculated (Figure 2B). We examine how the protein molecules and subunit interface interact with organic solvents by molecular dynamic simulations (MDS), which offers a molecular-level window into organic solvent tolerance and guides the design of novel enzymes. It can also enable a new type of reaction-solvent systems that are easily adoptable to numerous biosynthesis routes.

### 4.3. Conservation and Co-Evolution Analysis

Co-evolved amino acid residues are groups of residues where mutations appear simultaneously during the billions of years evolution [40]. Co-evolution occurs when the interaction between two or more residues is crucial for a protein’s stability or functionality. There are relative conserved interaction networks of amino acid residues networks that modulate the discrete functional properties in an enzyme superfamily [41]. Incorporating the evolutionary information of an enzyme family can continue to push forward the studies of the organic solvent tolerance of oxidoreductases [42]. The characterization of the conservation the interfacial binding residues, catalytic residues, and their functions, is key to understanding the impact of mutations in evolution, and further the enzyme design [43]. Enzyme family evolution provide a mechanistic perspective of highlighting common mechanisms that have been observed across multiple examples. 

Blanquart et al. studied the evolutionary route of malate dehydrogenases (MalDH) within *Halobacteria* by monitoring the stability of various MalDHs. Oligomeric states and enzymatic properties were studied as a function of the concentration for different salts. The result indicated a variety of evolutionary processes, such as amino acid residues replacement, and gene duplication and replacement resulted in significant differences in solubility, stability, and catalytic properties between these enzymes in the three *Halobacteriales, Haloferacales*, and *Natrialbales* orders since the LCAHa MalDH [44]. The result indicated how a stability trade-off favors the emergence of new properties during adaptation to organic solvents into a new view of halophilic protein adaptation. The dynamic conformation change in organic solvents can be mediated by energy transduction networks of coupling residues by evolution [45]. A distinct amino acid interaction network encounters different evolutionary bottlenecks. New technology allows scientists to create enzyme functions in the laboratory using evolutionary principles in months rather than millennia. Therefore, different mechanisms emerge along evolutionary trajectories toward improved organic solvents tolerance.

**Figure 3:**
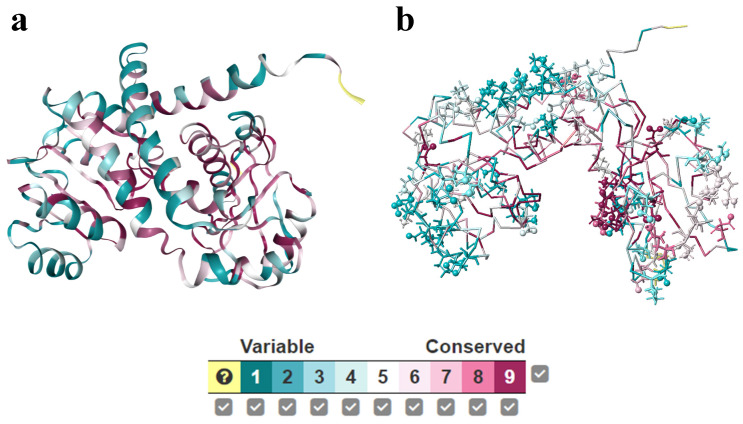
Conservation analysis of phenylalanine dehydrogenase from *Natranaerobius thermophilus*, (**a**) Conservation analysis of all amino acid residues; (**b**) conserved salt bridges.

A small part of amino acids (typically 10–30%) show strong mutual coevolution, which also comprise spatially distributed but structurally continuous subnetworks [46]. The coevolution analysis can be narrowed down to the range of key residues for mutation selection [47]. These findings are elucidating enzyme evolutionary trajectories and offer a promise for the design and engineering of artificial enzymes. Conservation analysis of phenylalanine dehydrogenase from *Natranaerobius thermophilus* was carried out by ConSurf web server (Figure 3a) and further conserved salt bridges were identified (Figure 3b) [48]. Molecular dynamic simulation results indicated that conserved salt bridges contribute to the stability of enzymes. Molecular mechanisms underlying the organic solvents adaptation of enzyme catalysis is characterized by evolution analysis. Evolution solved the enzyme’s key kinetic obstacle, which is how to maintain catalytic speed by exploiting the transition-state heat capacity in a non-aqueous system.

## 5. Design of Oxidoreductases with High Organic Solvents Tolerance 

Expanding the synthetic capabilities to apply enzymes in a non-aqueous system is a dream for protein engineers and organic chemists. However, the general and transferable design principles to improve the organic solvents resistance of enzymes are poorly understood. The study indicated that organic solvent tolerance is greatly dependent on the whole structure of the halophilic enzyme, which resulted from million years of evolution. Therefore, it may be one of the reasons as to why improving organic solvent tolerance by protein engineering strategies such as directed evolution and site-mutations is difficult [49]. 

The development of new efficient computational protocols to design an active enzyme for a given reaction would reduce the experimental costs [50]. Organic solvents tolerant to dehydrogenases form natural diversity, which reveals two design strategies, including surface charge engineering and substrate binding pocket engineering [49]. Strengthening protein surface hydration via regulating the surface charge is an effective and efficient strategy for tailoring enzyme stability and improving their resistance in organic solvents [51]. It is found that buried waters contribute most to rapid equilibration in organic solvents, with slow-diffusing waters giving similar results [52]. Enzyme gates are key residues in the substrate tunnel that control reversible conformational changes. Gate keeper residues affect the accessibility of solvent molecules to the binding pocket. Cheng et al. identified the beneficial residues at the “enzyme gate” region via computational analysis, alanine scanning, and site-saturation mutagenesis. ω-amine transaminase variants (*Ac*ATA_M2_) *Ac*ATA_M2_^F56D^, and *Ac*ATA_M2_^F56V^, not only displayed improved enzyme activity but also showed enhanced DMSO resistance. The half-life value increased from 25.71 to 42.49 h under 60% DMSO. MDS revealed that the increase in DMSO resistance was mainly caused by the decrease in the number of DMSO molecules in the substrate-binding pocket [53]. 

Musa et al. [54] studied the tune of substrate scope of secondary ADHs from *T. pseudoethanolicus* and *T. brockii* by using site-directed mutagenesis and directed evolution for chiral alcohols and amines in organic solvents. Munawar et al. [55] studied the glutamate dehydrogenase (GDH) from *Halobacterium salinarum,* which was converted into a dehydrogenase accepting L-methionine, L-norleucine, and L-norvaline as substrates by site-directed mutation and showed enhanced thermostability and organic solvent tolerance even at 70 °C. Javier et al. [56] constructed mutant libraries by random mutagenesis and in vivo DNA shuffling. The final variant of this evolutionary campaign carried 9 mutations that enhanced its activity in the presence of 30% acetonitrile (*v*/*v*) up to 23-fold over parental type, which was also stable in acetone, methanol, and dimethyl sulfoxide mixtures. Li et al. [57] studied the interactions between eight GDH mutants and NADP^+^, which showed that the enzyme activity of mutant P45A was 1829 U/mg, with an improvement of 28-fold compared to wild-type GDH. P45A also showed better catalytic ability in 10% isopropanol, which was 19-fold that of the wild-type GDH. Rosli at al. [58] reported that *A. geothermalis* ALDH can maintain an activity level of more than 50% in the 25% of DMSO, methanol, xylene, *n*-hexane, octanol, 2-propanol, *n*-heptane, and *n*-tetradecane.

Current strategies to identify enzyme activity rely on laborious tests carried out by incubation in different organic solvents and determination of residual activity. Computational techniques can be used to engineer enzymatic reactivity, substrate specificity and ligand binding, access pathways and ligand transport, and global properties such as protein stability, solubility, and flexibility [59]. To design organic solvent resistant enzymes with reduced time and screening effort in lab experiments, motifs assembly strategy was developed. Wang et al. reported the motifs of halophilic and thermophilic malate dehydrogenases (MDHs), which were identified by their adaptive response to a high salt concentration. Follow by motifs reassembly, novel MDHs were obtained with computational and experimental valuation. The results indicated that the interfacial interactions of dimer play a key role in MDH activity and stability [60]. A molecular understanding of functional charged networks alters the enzyme conformational landscape, which affects the stability. To understand the effect on enzyme stability of exposure to such non-natural conditions, we used the designed experiments to mimick industrial conditions and complemented them with a suite of analytical characterization tools.

Identifying the specific mechanisms that need to be improved when tailoring the enzyme structure considerably improves the chances to succeed in generating highly efficient and robust oxidoreductases in the future. 

## 6. Conclusions and Outlook

Improving enzyme activity and stability for non-aqueous catalysis is crucial for biocatalysis. Extreme enzymes play key roles in catalyzing valuable chemicals effectively and making them environmentally friendly. In this study, we focused on the mining and modification of robust oxidoreductases from extremophiles to meet the increasing demand of biocatalysis in non-aqueous systems [61]. We integrated gene mining, molecular dynamics, and co-evolutionary analysis for reliable target prediction and regulation of the extreme oxidoreductases for the biocatalysis in non-aqueous systems. The dominant effects of organic solvents on enzyme structure and function, and the potential of solvent and protein engineering to design enzymes to function optimally in organic media were discussed.

The challenges and future prospects for extreme enzymes in non-aqueous systems are discussed below:

1. A current challenge is how metagenomic mining can improve the discovery of polyextremophilic enzymes from multiple sources. Biotechnological exploitation of oxidoreductases from sequence derived statistics to the detailed atomic-level modeling of enzymes can provide cutting-edge technology in sequence annotation pipelines and bioinformatics resources;
2. Using machine learning to effectively analyze big data on a higher-dimensional levels to establish an exemplary discipline assist experiment and reveal the interrelationships between the gene data, structure, and function [62];
3. Key research challenges focus on high throughput screening and for the expression of extreme enzymes through dedicated hosts and purification systems and upscaling;
4. Using extreme enzymes for electrochemical reaction offers enhanced stability and applications in extreme environments where common oxidoreductase would not survive [63]. The development of organic phase enzyme electrodes (OPEEs) is essential for the detection of various hydrophobic substrates in organic media for a variety of health and industrial applications [64,65];
5. Novel immobilization strategy for oxidoreductases by tailoring the enzyme microenvironment for the further enhancement of organic solvent tolerance, such as MOF and polymeric microcapsules as the immobilization carrier [66,67].

Other innovative research in the field and future outlooks on the highly efficient oxidoreductases in a non-aqueous reaction system have received considerable attention for their potential application in biosynthesis and for bridging the gap between laboratorial and industrial production [68].

## Figures and Tables

**Table 1:** Dehydrogenase in the reaction system with organic solvents.

Enzyme	Reaction System	Substrate and Products	Cofactor	Activity	Ref.
HeADH-II from *Halomonas elongata*,coupled with a NADH-oxidase from *Lactobacillus pentosus* (LpNOX)	10–20% DMSO	Cinnamaldehyde to cinnamyl alcohol	NAD^+^	92% activity in 10% (*v*/*v*) DMSO 82% in 20% (*v*/*v*) DMSO	[22]
Alcohol Dehydrogenase from the Atlantis II Deep Red Sea brine pool	10% (*v*/*v*) acetonitrile, methanol, and DMSO	Cinnamyl-methyl-ketone to aliphatic alcohols; raspberry ketone to aromatic alcohols	NADH	70–80% activity after 32 h in 10% (*v*/*v*) acetonitrile, methanol, and DMSO	[23]
Halophilic alcohol dehydrogenase (ADH2) from *Haloferax volcanii*	DMSO and methanol	Alcohol to acetaldehyde	NAD^+^	47% activity in 30%DMSO 38% activity in 30% MeOH after 72 h	[10]
Formate dehydrogenase from *Burkholderia dolosa* PC543 (BdFDH)	DMSO, acetonitrile, methanol, ethanol, isopropanol	Sodium formate to carbon dioxide	NAD(P)^+^	170% activity in 30% DMSO	[24]
Glucose-1-dehydrogenase (GDH) from *Paenibacillus pini*	10% *v*/*v* or 50% *v*/*v* of DMSO	Glucose to glucono-δ-lactone	NAD^+^	90% activity in 10% *v*/*v* DMSO; 80% activity in 50% *v*/*v* DMSO	[25]
Glutamate dehydrogenase from *Halobacterium salinarum* strain NRC-36014	Methanol, ethanol, acetone, acetonitrile, 2-propanol, DMSO and DMSF	Glutamate to α-ketoglutarate	NADH/NAD^+^	100% activity 10% DMSO; more than 80% activity in 10% MeOH and 10% acetonitrile	[26]
Type II flavin-containing monooxygenase (FMO-E) and horse liver alcohol dehydrogenase (HLADH) as a bifunctional fusion enzyme	MTBE and CPME	Cyclobutanone and butane-1,4-diol to γ-butyrolactone	NAD+/NADH	95 % (*v*/*v*) organic solvent, and 5 % (*v*/*v*) aqueous buffer 0.5 mM NAD+	[27]

## Data Availability

Not applicable.

## Abbreviations

CPME	Cyclopentyl methyl ether
MOF	Metal-organic frameworks
MDS	Molecular dynamic simulation
DMSO	Dimethyl sulfoxide
MTBE	Methyl tert-butyl ether
